# Treatment of optic neuritis with erythropoietin: impact on white matter and optic nerve MRI characteristics

**DOI:** 10.1093/braincomms/fcag082

**Published:** 2026-03-19

**Authors:** Sebastian Küchlin, Niklas Lützen, Navid Farassat, Ricarda Diem, Philipp Albrecht, Orhan Aktas, Christoph Heesen, Amelie Pielen, Horst Urbach, Martin J Hug, Kurt-Wolfram Sühs, Wolf A Lagrèze

**Affiliations:** Eye Center, Medical Center—University of Freiburg, Faculty of Medicine, University of Freiburg, D-79106 Freiburg, Germany; Department of Neuroradiology, Medical Center—University of Freiburg, Faculty of Medicine, University of Freiburg, D-79106 Freiburg, Germany; Eye Center, Medical Center—University of Freiburg, Faculty of Medicine, University of Freiburg, D-79106 Freiburg, Germany; Department of Neurology and National Center for Tumor Diseases, Faculty of Medicine, University Hospital Heidelberg, D-69120 Heidelberg, Germany; Department of Neurology, Maria Hilf Clinics Mönchengladbach, D-41063 Mönchengladbach, Germany; Department of Neurology, Medical Faculty, Heinrich Heine-Universität Düsseldorf, D-40225 Düsseldorf, Germany; Department of Neurology, Medical Faculty, Heinrich Heine-Universität Düsseldorf, D-40225 Düsseldorf, Germany; Institute of Neuroimmunology and Multiple Sclerosis and Department of Neurology, University Medical Center Hamburg-Eppendorf, D-20246 Hamburg, Germany; Maximilians-Augenklinik GmbH, D-90491 Nürnberg, and University Clinic for Ophthalmology, Hannover Medical School, D-30625 Hannover, Germany; Department of Neuroradiology, Medical Center—University of Freiburg, Faculty of Medicine, University of Freiburg, D-79106 Freiburg, Germany; Pharmacy, Medical Center—University of Freiburg, Faculty of Medicine, University of Freiburg, D-79106 Freiburg, Germany; Department of Neurology, Hannover Medical School, D-30625 Hannover, Germany; Eye Center, Medical Center—University of Freiburg, Faculty of Medicine, University of Freiburg, D-79106 Freiburg, Germany

**Keywords:** optic neuritis, multiple sclerosis, neuroprotection, erythropoietin

## Abstract

We previously conducted the TONE study (Treatment of optic neuritis with erythropoietin, NCT01962571), which analysed 103 patients with acute optic neuritis and no prior history of multiple sclerosis after randomization to receive high-dose erythropoietin or placebo as an adjunct to methylprednisolone pulse therapy. All visual system-related outcomes yielded negative results. The goal of the current investigation is to assess whether erythropoietin administration influenced inflammatory optic nerve and cerebral lesions or optic nerve atrophy on MRI. We determined the sensitivity of MRI in detecting optic nerve lesions and assessed the influence of the methylprednisolone treatment delay.

In this ancillary analysis of MRI images that were acquired during the TONE trial, we determined the optic nerve cross-sectional area, the extent of optic nerve T2 and gadolinium-enhancing lesions, the number of T2 and gadolinium-enhancing juxtacortical, periventricular and infratentorial white matter lesions, and the fulfilment of radiological dissemination-in-space and dissemination-in-time (2017 McDonald criteria). Lesion changes were analysed by non-parametrical statistical methods (Wilcoxon rank sum test and chi-square test) and the time to the occurrence of any new cerebral lesion was analysed by a Cox proportional hazards model. The impact of the time to methylprednisolone treatment was investigated through stratified analyses with a median split.

We obtained baseline MRI from 77/103 patients (75%), six months’ follow-up from 40/103 patients (39%), and included 52/103 patients (50%) in the time-to-event analysis. At baseline, gadolinium-enhancing optic nerve lesions were more frequent in the placebo compared to the erythropoietin group. Otherwise, baseline characteristics were balanced. We found no clinically meaningful or statistically significant differences between erythropoietin and placebo recipients in terms of lesion changes or optic atrophy. The sensitivity for detecting any optic nerve lesion was 82% at baseline and 85% at month 6. Outcomes were similar between patients whose methylprednisolone treatment was initiated early (<6 days) versus late (≥6 days) after symptom onset.

In conclusion, this study provides class II evidence that high-dose erythropoietin in conjunction with methylprednisolone has no effect on the evolution of optic nerve or cerebral white matter lesions in patients presenting with acute optic neuritis.

## Teaser

Küchlin *et al*. report that high-dose erythropoietin had no statistically significant effect on the evolution of MRI optic nerve lesions, cerebral white matter lesions or optic nerve atrophy when given as an adjunct to methylprednisolone for acute optic neuritis.

## Graphical Abstract

**Graphical Abstract:**
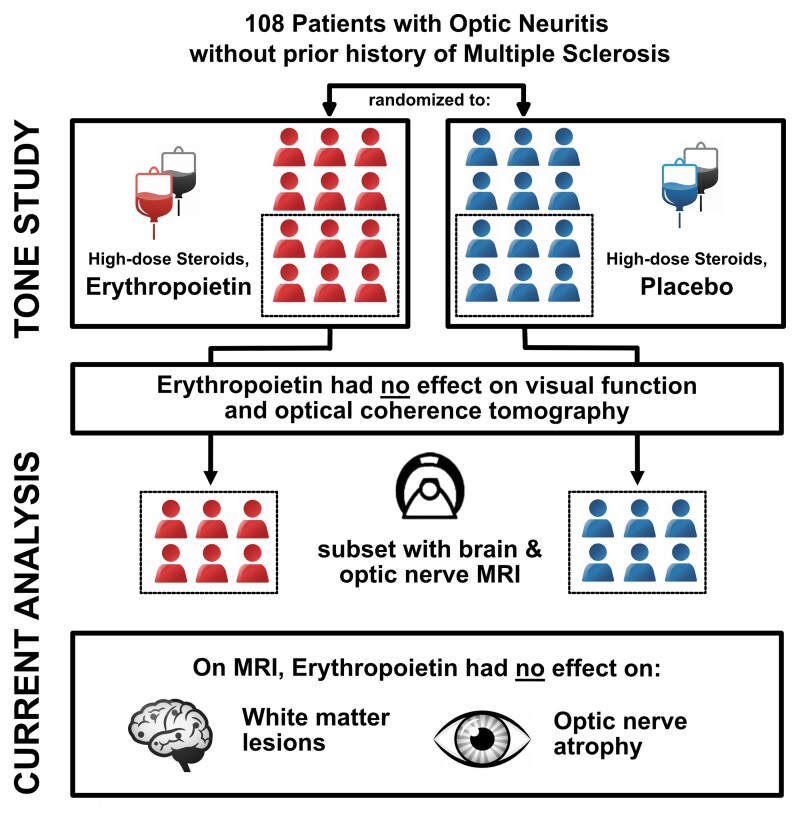

## Introduction

Acute idiopathic and multiple sclerosis (MS)-associated optic neuritis (ON) is the most prevalent optic neuropathy in young adults, resulting in the loss of retinal ganglion cells.^1^ It serves as a model disease for MS and is commonly used in visual pathway neuroprotection trials.^2^ Currently, the sole guideline-based treatment is the administration of high-dose glucocorticosteroids such as methylprednisolone (MP), which accelerates visual recovery but has no proven impact on long-term outcomes.^3,4^ Based on a growing body of pre-clinical and clinical evidence for neuroprotective effects of erythropoietin (EPO),^5-7^ we conducted the TONE trial (Treatment of patients with optic neuritis with erythropoietin), which randomized 108 patients with a first episode of acute ON but without known MS to either high-dose EPO (33000 IU/day for 3 days) or placebo (saline solution) as an adjunct to methylprednisolone pulse therapy (1000 mg/d for three days). The trial’s main outcome was at 6 months, with an open-label follow-up at 24 months. It was designed and powered for visual system outcomes, which yielded negative results.^8,9^ However, the rate of conversions from clinically isolated syndrome to MS was higher in the placebo compared to the EPO group over 6 months [hazard ratio (HR): 1.91; 95% CI: 1.06–3.44; *P* = 0.03]^8^ but not over the long-term follow-up of 24 months (*P* = 0.07).^9^

The aim of this work is to assess whether treatment with EPO influenced optic nerve atrophy and optic nerve or cerebral white matter lesion changes on MRI. Following recent efforts to minimize the time to treatment with glucocorticosteroids in patients with MS-associated or idiopathic ON,^10^ we also conducted post hoc analyses comparing early versus late treatment. Finally, we assessed the sensitivity of orbital MRI for the detection of optic nerve lesions in a pooled analysis.

## Materials and methods

### Study design

We retrospectively collected MRI scans from patients enrolled in the TONE study cohort to assess cerebral optic nerve and white matter lesions potentially related to a chronic inflammatory disease. Participants of the TONE trial were recruited from 12 German academic centres between 25 November 2014 and 9 October 2017. The main inclusion criteria were the following (i) a first-time occurrence of a unilateral episode of acute optic neuritis without known history of multiple sclerosis, presenting within 10 days of symptom onset (decreased vision or pain/discomfort on eye movements), (ii) a high contrast visual acuity < 0.5 decimal (3/6 Snellen) and (iii) a negative test for aquaporin-4 serum antibodies. The trial randomized a total of 108 patients, 103 of whom were included in the full analysis set according to the statistical analysis plan. The reasons for exclusion from the primary analysis were one withdrawn consent, two loss to follow-up, one not receiving the allocated study medication and one revised diagnosis.

### Acquisition and analysis of MRI data

MRI was not an obligatory part of the TONE study protocol due to budget constraints^11^ but recommended for all patients at baseline and month 6. Therefore, MRI acquisition protocols and scanners varied (details provided in the results section). All MRIs were evaluated by a neuroradiologist (N.L.) with more than 10 years of experience who was blinded to both the treatment assignment and the laterality of optic neuritis (left eye or right eye). We calculated the extent of T2-weighted and T1-weighted Gadolinium (Gd)-enhancing optic nerve lesions in mm as the product of the number of affected slices and slice thickness. In cases where coronal sequences were unavailable or of insufficient quality, we measured lesion lengths on axial sequences if available. We determined the optic nerve cross-sectional area on a single representative coronal retrobulbar section per eye. We recorded the number of infratentorial, juxtacortical and periventricular T2-weighted and T1-weighted Gd-enhancing lesions and assessed whether the radiological criteria for dissemination in time and dissemination in space in accordance with the International 2017 McDonald criteria^12^ were fulfilled.

### Statistical analysis

We performed our statistical analysis and data visualization in the R statistical programming language (version 4.3.2) with the RStudio interface and packages *tidyverse*, *gtsummary, survival* and *survminer*. The radiological software was DeepUnity Diagnost (version 1.2.0.3., DH Healthcare GmbH, Bonn, Germany). We used non-parametric statistical tests to analyse data that were not event based: Wilcoxon rank sum test for continuous data and Pearson’s χ^2^ test for categorical data. We analysed event-based data (survival without any new cerebral white matter lesions) with a Kaplan–Meier plot and a Cox proportional hazard model without adjustments, censoring survival times after 1 year. We considered two-sided *P* values < 0.05 as statistically significant and did not perform adjustments for multiple testing. Our report follows the STROBE guidelines.^13^

### Standard protocol approvals, registrations and patient consents

The TONE study was approved by the ethics committee of the University of Freiburg, Germany and the institutional review boards of all participating sites. All participants provided written informed consent.

## Results

### Availability of MRI data and scan characteristics

We obtained and analysed a total of 135 scans from 79 patients, which had been conducted at five centres, representing 77% of the TONE study population. Scan protocols were heterogeneous; these are described in detail in Supplementary Table 1.

### Baseline characteristics

Baseline MRI scans were available from 77 patients, of whom 40 had received EPO and 37 had received placebo. The overall characteristics of the treatment groups were similar, shown in Table 1 and Supplementary Table 2. Gadolinium enhancing optic nerve lesions were present less frequently in the EPO (17/32, 53%) versus the placebo group (23/29, 79%; χ^2^ = 4.07, *P* = 0.04). Gadolinium enhancing and T2-weighted optic nerve lesion lengths were similar between groups. In the subset of 40 patients for whom a follow-up MRI at month 6 was available, the frequency of optic nerve MRI lesions was balanced, with otherwise similar baseline characteristics (Table 1). The number of cerebral white matter lesions (T2 or Gadolinium-enhancing) was low in most patients and similar between groups (Supplementary Table 2).

**Table 1:** Demographic and baseline optic nerve MRI characteristics

	All patients with MRI at baseline				Subset with follow-up at month 6			
Characteristic	*N*	EPO *N* = 40^a^	PLACEBO *N* = 37^a^	Statistic, *P* value^b^	*N*	EPO *N* = 20^a^	PLACEBO *N* = 20^a^	Statistic^b^, *P* value^b^
*Demographics*								
Sex	77			χ^2^ = 0.23, *P* = 0.63	40			χ^2^ = 0.00, *P* = 1.00
Female		29 (72%)	24 (65%)			14 (70%)	13 (65%)	
Male		11 (28%)	13 (35%)			6 (30%)	7 (35%)	
Age, years	77	28.5 (24.8–34.3)	31.0 (27.0–37.0)	W = 625.0, *P* = 0.24	40	28.5 (25.0–31.5)	30.0 (27.0–37.8)	W = 172.5, *P* = 0.50
*Optic Nerve MRI*								
Cross-sectional area, mm^2^	55	6.0 (4.5–7.0)	5.0 (4.0–8.0)	W = 403.5, *P* = 0.60	30	6.0 (4.5–7.5)	5.0 (4.0–6.5)	W = 138.0, *P* = 0.29
Cross-sectional area, inter-eye ratio	54	1.0 (0.9, 1.0)	1.0 (1.0, 1.1)	W = 316.5, *P* = 0.32	30	1.0 (0.8–1.2)	1.0 (1.0–1.0)	W = 105.0, *P* = 0.75
T2 lesions								
Lesion present	70	25 (69%)	27 (79%)	χ^2^ = 0.46, *P* = 0.50	36	15 (88%)	15 (79%)	χ^2^ = 0.09, *P* = 0.77
Lesion length, mm	52	9.0 (6.0–12.0)	9.0 (6.0–12.0)	W = 299.0, *P* = 0.47	30	9.0 (6.0–9.0)	9.0 (9.0–12.0)	W = 84.5, *P* = 0.23
Gd + lesions								
Lesion present	62	17 (52%)	23 (79%)	χ^2^ = 4.07, ***P*=** 0**.04**	35	10 (59%)	12 (67%)	χ^2^ = 0.02, *P* = 0.90
Lesion length, mm	40	9.0 (6.0–12.0)	9.0 (6.0–10.5)	W = 193.5, *P* = 0.97	22	9.0 (6.8–9.0)	9.0 (6.0–9.8)	W = 56.5, *P* = 0.80

Gd+, Gadolinium contrast enhancing.

^a^n (%); median (IQR).

^b^Pearson's chi-squared test; Wilcoxon rank sum test.

### Optic nerve characteristics at month 6

We found no statistically significant differences between the EPO and placebo groups for any of the optic nerve characteristics (Table 2).

**Table 2:** Optic nerve MRI characteristics at month 6, by treatment group

Characteristic	*N*	EPO *N* = 20^a^	PLACEBO *N* = 20^a^	Statistic, *P* value^b^
All patients				
Cross-sectional area, mm^2^	25	5.50 (4.00, 7.00)	4.00 (4.00, 5.00)	W = 103.0, *P* = 0.14
Change in cross-sectional area, mm^2^	21	−1.00 (−1.00, 0.00)	−1.00 (−1.00, 0.00)	W = 49.5, *P* = 0.76
Cross-sectional area, inter-eye ratio	24	0.87 (0.80, 1.00)	0.90 (0.78, 1.00)	W = 75.5, *P* = 0.76
Patients with T2 lesions at baseline		*N* = 15	*N* = 15	
T2 lesion present	25	12 (92%)	10 (83%)	χ^2^ = 0.01, *P* = 0.94
T2 lesion length, mm	25	9.0 (6.0, 9.0)	9.0 (5.3, 15.8)	W = 49.0, *P* = 0.47
Change in T2 lesion length, mm	25	0.0 (0.0, 3.0)	0.0 (−6.0, 3.0)	W = 84.0, *P* = 0.76
Patients with Gd + lesion at baseline		*N* = 10	*N* = 12	
Gd + lesion present	15	4 (57%)	2 (25%)	χ^2^ = 0.55, *P* = 0.46
Gd + lesion length, mm	6	9.0 (8.3, 11.3)	4.5 (3.8, 5.3)	W = 7.5, *P* = 0.15
Change in Gd + lesion length, mm	15	−6.0 (−7.5, 0.0)	−8.0 (−12.0, −6.0)	W = 37.0, *P* = 0.12

Gd+, Gadolinium contrast enhancing.

^a^ *n* (%); median (IQR).

^b^Pearson's chi-squared test; Wilcoxon rank sum test.

**Figure 1:**
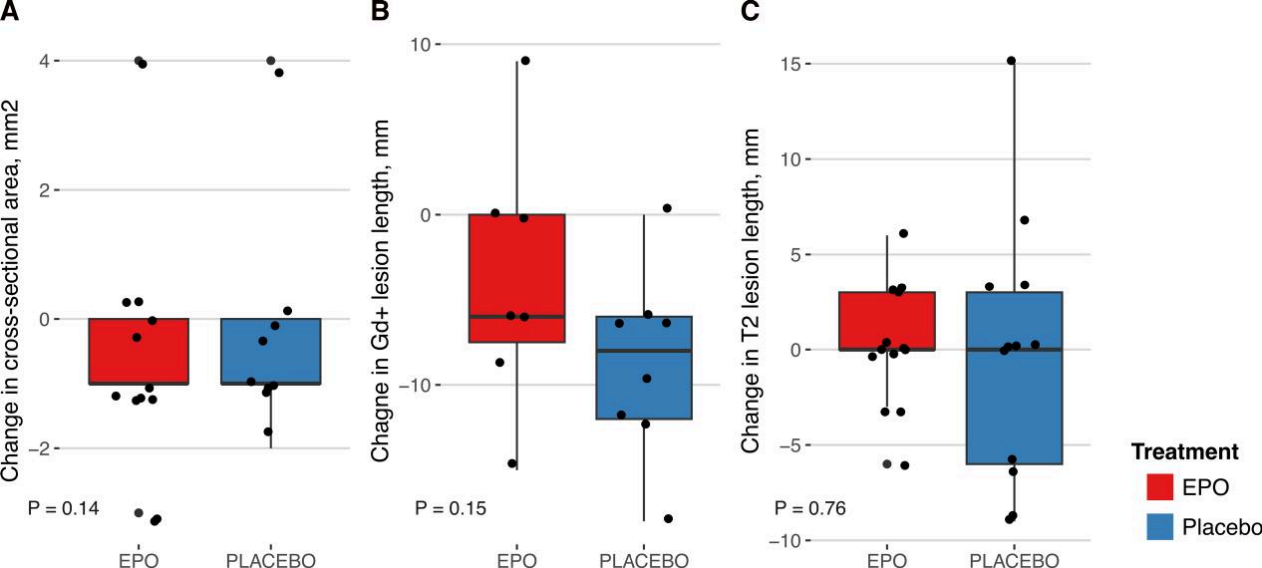
**Outcomes of optic nerve MRI lesions at month 6.** (**A**) Change in optic nerve cross-sectional area. (**B**) Change in Gadolinium-enhancing lesion length. *P* values were calculated by the Wilcoxon rank sum test. (**C**) Change in T2 lesion length. Points are individual patient measurements. Boxes extend from the 25th to the 75th percentile (Q1 to Q3), thick horizontal lines denote the median, and whiskers extend to the most extreme datapoint that is no more than 1.5 interquartile range from the box hinges. *Gd+ = Gadolinium enhancing*.

The median cross-sectional area of the optic nerve was slightly higher in the EPO compared to the placebo group [median: 5.50 mm^2^ (IQR: 4.00–7.00) versus 4.00 mm^2^ (4.00–5.00), W = 103.0, *P* = 0.14]. The median change, corresponding to optic nerve atrophy, was −1.00 mm^2^ (−1.00 to 0.00) in both groups (W = 49.5, *P* = 0.76, Fig. 1A).

In patients who had Gd + lesions at baseline, there was a tendency towards longer Gd + lesion lengths at month 6 in the EPO versus the placebo group [median: 9.0 (8.3–11.3) versus 4.5 (3.8–5.3), *W* = 7.5, *P* = 0.15], while the median change was slightly more pronounced in the placebo group [EPO: −6 mm (−7.5 to 0.0) versus placebo: −8.0 (−12.0 to −6), *W* = 37.0, *P* = 0.12] (Fig. 1B). In patients who had T2 optic nerve lesions at baseline, both the median T2 lesion length and the length change at six months were similar between treatment groups [EPO: 9.0 (6.0–9.0) versus placebo: 9.0 (5.3–15.8), *W* = 49.0, *P* = 0.47 and 0.0 (0.0–3.0) versus 0.0 (−6.0 to 3.0), *W* = 84.0, *P* = 0.76, respectively] (Fig. 1C).

### Pooled analysis of MRI sensitivity for optic nerve lesions

At baseline, T2 optic nerve lesions in the affected eye were detected in 52/70 patients (74%) and T1 Gd+ lesions were detected in 40/62 patients (65%). The overall sensitivity for any optic nerve lesion (T2 or T1 Gd+) at baseline was 82% (detected in 54/66 patients).

At month 6, T2 optic nerve lesions were detected in 26/32 patients (81%), T1 Gd+ lesions were detected in 7/29 patients (24%). Any optic nerve lesion (T2 or T1 Gd+) was present in 85% of patients (28/33) (Table 3).

**Table 3:** Pooled sensitivity analysis for the detection of optic nerve lesions on MRI

	Baseline		Month 6	
Characteristic^a^	*N*		*N*	
Any optic nerve lesion present	66	54 (82%)	33	28 (85%)
T2 optic nerve lesion present	70	52 (74%)	32	26 (81%)
Gd + optic nerve lesion present	62	40 (65%)	29	7 (24%)

Gd+, Gadolinium contrast enhancing.

^a^ *n* (%).

### Change in cerebral white matter lesion number at month 6

**Figure 2:**
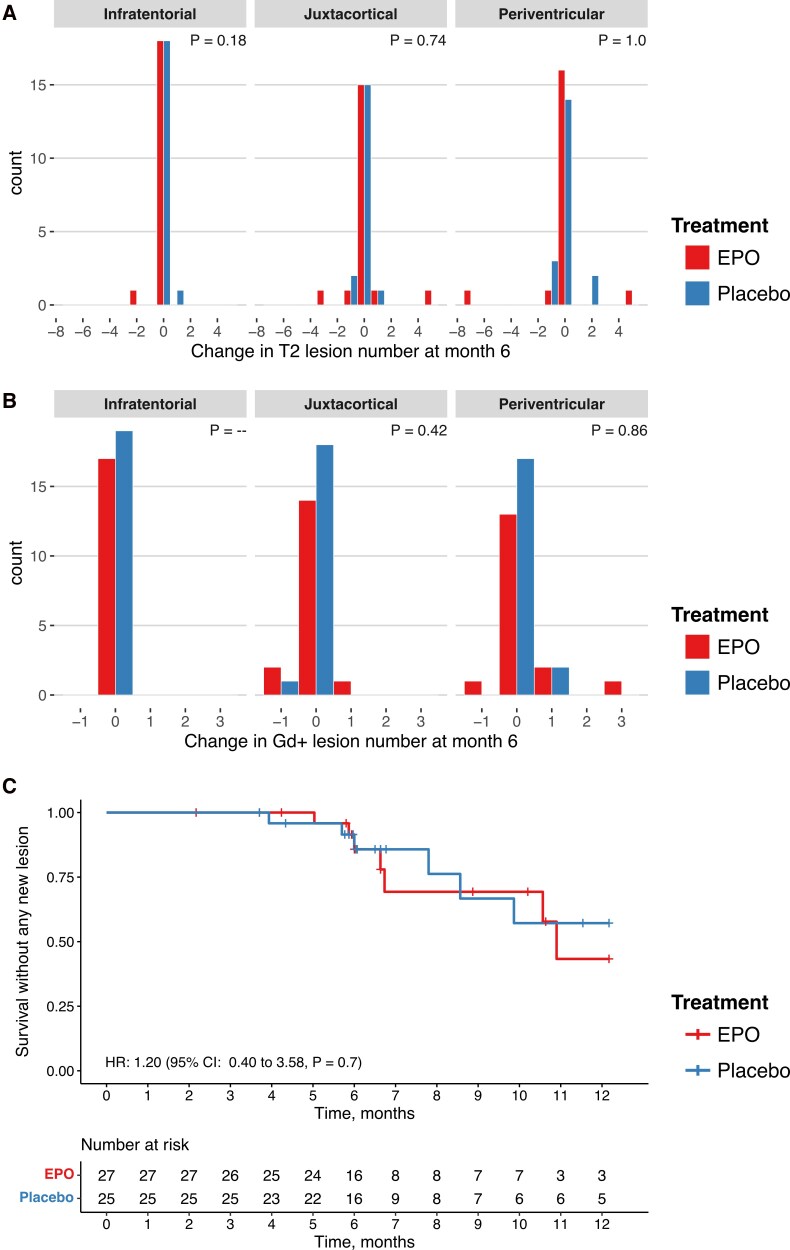
**Outcomes of cerebral MRI lesions.** (**A**) Change in T2 enhancing lesion counts at month 6, by treatment group. Statistical analysis was by a Wilcoxon rank sum test (Infratentorial: *N* = 38, *W* = 162.0, *P* = 0.18; Juxtacortical: *N* = 37, *W* = 179.0, *P* = 0.74; Periventricular: *N* = 38, *W* = 180.5, *P* = 1.0). (**B**) Change in Gadolinium enhancing lesion counts at month 6, by treatment group. Statistical analysis was by a Wilcoxon rank sum test (Infratentorial: *N* = 36, *W* = 161.5, *P* value could not be calculated; Juxtacortical: *N* = 36, *W* = 160.0, *P* = 0.95; Periventricular: *N* = 36, *W* = 165.5, *P* = 0.86). (**C**) Survival without any new central white matter lesion. Vertical lines depict censored data. Statistical analysis was by a Cox proportional hazards model. Gd+ = Gadolinium enhancing.

Lesion counts remained mostly stable in both treatment groups, with the most dynamic changes observed in periventricular lesions. Overall, there were no significant differences between the EPO and placebo groups (Fig. 2A and B, Table 4).

**Table 4:** Cerebral MRI characteristics at month 6, by treatment group

Characteristic^a^	*N*	EPO *N* = 20^a^	PLACEBO *N* = 20^a^	Statistic, *P* value^b^
Change in number of T2 infratentorial lesions	38			χ^2^ = 2.00, *P* = 0.37
−1 to −3		1 (5.3%)	0 (0%)	
0		18 (95%)	18 (95%)	
1–3		0 (0%)	1 (5.3%)	
Median (IQR)		0 (0,0)	0 (0,0)	W = 162.0, *P* = 0.18
Change in number of T2 juxtacortical lesions	37			χ^2^ = 0.97, *P* = 0.81
−1 to −3		2 (11%)	2 (11%)	
0		15 (79%)	15 (83%)	
1–3		1 (5.3%)	1 (5.6%)	
4–6		1 (5.3%)	0 (0%)	
Median (IQR)		0 (0,0)	0 (0,0)	W = 179.0, *P* = 0.74
Change in number of T2 periventricular lesions	38			χ^2^ = 5.13, *P* = 0.27
≤ −7		1 (5.3%)	0 (0%)	
−1 to −3		1 (5.3%)	3 (16%)	
0		16 (84%)	14 (74%)	
1–3		0 (0%)	2 (11%)	
4–6		1 (5.3%)	0 (0%)	
Median (IQR)		0 (0,0)	0 (0,0)	W = 180.5, *P* = 1.0
Change in total number of T2 lesions	37			χ^2^ = 2.55, *P* = 0.64
≤ −7		1 (5.3%)	0 (0%)	
−1 to −3		2 (11%)	3 (17%)	
0		14 (74%)	13 (72%)	
1–3		1 (5.3%)	2 (11%)	
7+		1 (5.3%)	0 (0%)	
Median (IQR)		0 (0,0)	0 (0,0)	W = 169.0, *P* = 0.95
Change in number of Gd + infratentorial lesions	36	17 (100%)	19 (100%)	χ^2^ = 0.11, *P* = 0.74
Median (IQR)		0 (0,0)	0 (0,0)	W = 161.5, –^c^
Change in number of Gd + juxtacortical lesions	36			χ^2^ = 1.73, *P* = 0.42
−1 to −3		2 (12%)	1 (5.3%)	
0		14 (82%)	18 (95%)	
1		1 (5.9%)	0 (0%)	
Median (IQR)		0 (0,0)	0 (0,0)	W = 160.0, *P* = 0.95
Change in number of Gd + periventricular lesions	36			χ^2^ = 1.63, *P* = 0.44
−1 to −3		1 (5.9%)	0 (0%)	
0		13 (76%)	17 (89%)	
1–3		3 (18%)	2 (11%)	
Median (IQR)		0 (0,0)	0 (0,0)	W = 165.5, *P* = 0.86
Change in total number of Gd + lesions	36			χ^2^ = 1.76, *P* = 0.62
−1 to −3		1 (5.9%)	1 (5.3%)	
0		13 (76%)	17 (89%)	
1–3		2 (12%)	1 (5.3%)	
4–6		1 (5.9%)	0 (0%)	
Median (IQR)		0 (0,0)	0 (0,0)	W = 179.5, *P* = 0.39
MRI dissemination in time	40	6 (30%)	4 (20%)	χ^2^ = 0.13, *P* = 0.72
MRI dissemination in space	40	9 (45%)	11 (55%)	χ^2^ = 0.10, *P* = 0.75

Gd+, Gadolinium contrast enhancing.

^a^ *n* (%); Median (IQR).

^b^Pearson's chi-squared test; Wilcoxon rank sum test.

^c^ *P* value could not be calculated.

### Dissemination in time and space at month 6

There was no statistically significant difference in the fulfilment of radiological DIS and DIT criteria at month 6 between treatment groups (Table 4): Dissemination in time was given in 6/20 (30%) EPO and 4/20 (20%) placebo recipients (χ^2^ = 0.13, *P* = 0.72), while DIS was fulfilled in 9/20 (45%) and 11/20 (55%) patients in the EPO and placebo group, respectively (χ^2^ = 0.10, *P* = 0.75).

### Survival without any new cerebral white matter lesion

We found no clinically meaningful or statistically significant differences in the time to development of any new cerebral white matter lesion. The Kaplan–Meier curves for survival without any new (T2 or Gd+) cerebral white matter lesion followed similar trajectories between the EPO and placebo groups (Fig. 2C). The HR on Cox regression was 1.2 (95% CI: 0.40–3.58, *P* = 0.7) for EPO versus placebo treatment.

### Analysis of outcomes by MP treatment delay

As all patients received MP treatment, for this analysis, we pooled EPO and placebo recipients. We defined early MP treatment as a time from symptom onset to treatment initiation (treatment delay) < 6 days and late MP treatment as a treatment delay ≥ 6 days, based on a median split. Patients who received early treatment tended to be slightly younger [median age: 28.0 years (IQR: 23.0–37.0)] compared to patients with late treatment [30.0 (27.0–36.3), *W* = 599, *P* = 0.15]. The sex distribution and treatment assignment were similar between groups. There was a tendency towards a lower proportion of T1 Gd + optic nerve lesions detected at baseline in patients with early versus late treatment [lesion present in 17/30 patients (57%) versus 23/40 (72%), χ^2^ = 0.97, *P* = 0.33]. Otherwise, the baseline characteristics were similar between groups (Supplementary Table 3).

The outcomes of cerebral white matter MRI T2 lesion changes at month 6 are shown in Supplementary Table 4. The criteria for radiological dissemination in time were fulfilled more often in patients with early (7/20 [35%]) versus late treatment (3/10 [15%], χ^2^ = 1.20, *P* = 0.27). The change in Gd + enhancing periventricular lesions number at month 6 was higher in patients with early compared to late treatment (χ^2^ = 6.53, *P* = 0.04). Otherwise, outcomes were similar between groups.

New cerebral white matter lesions (T2 or Gd+) were detected at similar rates throughout the first 7 months, with a non-significant trend towards a higher rate in early treated patients thereafter (Supplementary Fig. 1). The HR on unadjusted Cox regression for early versus late treatment was 2.08 (95% CI: 0.67–6.42; *P* = 0.20).

Optic nerve MRI outcomes at month 6 are shown in Supplementary Table 5; these were similar between groups.

## Discussion

In this investigation, we performed a post hoc analysis of MRIs from a subset of patients who participated in the TONE trial. The goal was to assess the impact of EPO treatment on optic nerve and cerebral white matter lesions as well as optic nerve atrophy on MRI. Moreover, we assessed the sensitivity of MRI to detect optic nerve lesions and analysed the impact of early versus late MP treatment.

The prevalence of Gd enhancing optic nerve lesions at baseline was higher in the placebo group than in the EPO group (*P* = 0.04). Since extensive Gd-enhancing optic nerve lesions have been associated with worse outcomes in optic neuritis,^14^ this baseline imbalance could bias group comparisons. However, since the primary outcome was negative, such bias is unlikely to change the main conclusion. On follow-up, we found no meaningful group differences in either the absolute values or change characteristics of Gd-enhancing lesion lengths, T2 lesion lengths, or the optic nerve cross-sectional area. This was expected and confirms the negative results of the TONE trial in the visual system.

Regarding changes in cerebral white matter lesion counts, we found no clinically meaningful or statistically significant differences between EPO and placebo recipients. In light of this finding, we believe that the initial observation of lower rates of early conversions from clinically isolated syndrome to MS in EPO recipients made in the TONE study was more likely due to delayed workup than due to a treatment effect. This aligns with data from the VISION-PROTECT study, a smaller phase II randomized trial of EPO that used the same treatment protocol: Based on data from 35 patients, the authors reported more early conversions in placebo compared to EPO recipients, but found no difference in cerebral lesion count changes.^7^

One avenue of interest that has gained traction is the timing of glucocorticosteroid treatment in optic neuritis, as data from small case series have suggested a possible benefit of very early treatment.^10,15^ Comparing the upper and lower quantile of our cohort based on MP treatment delay with a split at 6 days, we found no evidence for a benefit of early treatment. An important potential source of confounding is that patients with more severe disease may present earlier, and treatment in these individuals may be initiated with greater haste. Nevertheless, our observations are consistent with previous analyses of visual outcomes in the TONE trial, which revealed no benefit of earlier treatment.^8,9^ A large prospective cohort (ACON) that investigates the timing of glucocorticosteroid treatment in optic neuritis is currently underway.^16^

With the recent inclusion of the optic nerve as a fifth dissemination-in-space area for the diagnosis of MS,^17^ we were interested in the sensitivity of orbital MRI to detect optic nerve lesions in our cohort. We found that optic nerve lesions could be detected in 82% at baseline and 85% at month 6, a higher percentage than in a recent MAGNIMS study^18^ but similar to a recent, large monocentric cohort.^19^ While our findings encourage the possibility of detecting optic nerve lesions months after a demyelinating event, they highlight that even in the presence of at least moderate visual impairment (all patients had initial visual acuity < 3/6 Snellen), a relevant proportion of optic nerve MRIs will be negative.

Our study has a number of limitations: The TONE trial was designed and powered for visual outcomes. MRI was encouraged but not mandatory, leading to substantial variability in protocols, scan quality, and data availability. An assessment of cerebral lesion sizes in addition to counts was determined not feasible due to this heterogeneity but might have brought additional insights. Confluent lesions were treated as single lesions, which may have underestimated the overall lesion numbers. Data on optic nerve lesion lengths should be interpreted cautiously due to high slice thicknesses. Our MRIs were evaluated by a single experienced neuroradiologist, who was however blinded to both the treatment assignment and to the laterality of the affected eye. Finally, the potential inclusion of patients with anti-myelooligodendrocyte glycoprotein (MOG)-associated ON represents a limitation inherent to the TONE study design, as MOG antibody testing was not universally performed.^8,9^

In conclusion, our analysis provides class II evidence that treatment with EPO does not impact optic nerve or cerebral white matter lesions in patients with acute idiopathic or MS-associated ON. This finding is consistent with the primary TONE trial results, which demonstrated no effect of EPO on a comprehensive battery of visual function tests.

## Supplementary Material

fcag082_Supplementary_Data

## Data Availability

Individual participant data, including data dictionaries, will be made available together with previous study data of the TONE trial to researchers with a methodologically sound proposal.
