# Supplementary material for: Treatment of optic neuritis with erythropoietin: impact on white matter and optic nerve MRI characteristics
Source: Brain Commun. 2026 Mar 19;8(2):fcag082. doi: 10.1093/braincomms/fcag082 (PMC13089568; doi:10.1093/braincomms/fcag082)
Supplement: fcag082_Supplementary_Data [file fcag082_supplementary_data.pdf]

Online-only supplement for:

Treatment of optic neuritis with erythropoietin: Impact on white matter and optic nerve MRI characteristics

Dr. Sebastian Küchlin[1]*, Dr. Niklas Lützen[2]*, Dr. Dr. Navid Farassat[1], Prof. Ricarda Diem[3], Prof. Philipp Albrecht[4], Prof. Orhan Aktas[5], Prof. Christoph Heesen[6], Prof. Amelie Pielen[7], Prof. Horst Urbach[2], Prof. Martin J Hug[8], Prof. Kurt-Wolfram Sühs[9], Prof. Wolf A Lagrèze[1]

*Sebastian Küchlin and Niklas Lützen contributed equally to this work.

Affiliations:

[1]Eye Center, Medical Center – University of Freiburg, Faculty of Medicine, University of Freiburg, Germany

[2]Department of Neuroradiology, Medical Center – University of Freiburg, Faculty of Medicine, University of Freiburg, Germany

[3]Department of Neurology and National Center for Tumor Diseases, Faculty of Medicine, University Hospital Heidelberg, Germany

[4]Department of Neurology, Maria Hilf Clinics Mönchengladbach, Germany

[5]Department of Neurology, Medical Faculty, Heinrich Heine-Universität Düsseldorf, Germany

[6]Department of Neurology and Institute of Neuroimmunology and Multiple Sclerosis, University Medical Center Hamburg-Eppendorf, Germany

[7]Maximilians-Augenklinik GmbH, Nürnberg, and University Clinic for Ophthalmology, Hannover Medical School, Germany

[8]Pharmacy, Medical Center – University of Freiburg, Faculty of Medicine, University of Freiburg, Germany

[9]Department of Neurology, Hannover Medical School, Germany

Supplementary Table 1: Characteristics of magnetic resonance imaging protocols.

**Characteristic**	**N (%)**
Assessment of cerebral Gd+ lesions	
Sequence	
MPRage, coronal	122 (90%)
T1 spin echo, axial	7 (5.2%)
Other	6 (4.4%)
Slice thickness	
1 mm	120 (92%)
2 mm	2 (1.5%)
3 mm	1 (0.8%)
5 mm	3 (2.3%)
6 mm	3 (2.3%)
Unknown/unavailable	5
Assessment of cerebral T2 lesions	
Sequence	
FLAIR, axial	121 (90%)
FLAIR, coronal	10 (7.4%)
Other	4 (3%)
Slice thickness	
1 mm	2 (1.5%)
3 mm	73 (54%)
5 mm	57 (42%)
6 mm	3 (2.2%)
Assessment of optic nerve cross-sectional area	
Sequence	
Short tau inversion recovery, coronal	47 (44.3%)
T2, coronal	45 (42.5%)
T2 fat saturated, coronal	14 (13.2%)
Unknown/unavailable	29
Slice thickness	
1 mm	1 (1.0%)
3 mm	98 (99%)
Unknown/unavailable	36
Assessment of optic nerve Gd+ lesions	
Sequence	
T1 spin echo, fat saturated, coronal	104 (99%)
T1 turbo spin echo, axial	1 (1%)
Unknown/unavailable	20
Slice thickness	
3 mm	104 (100%)
Unknown/unavailable	21
Assessment of optic nerve non contrast-enhancing lesions	
Sequence	
T2, fat saturated, coronal	55 (44%)
Short tau inversion recovery, coronal	55 (44%)
T2, axial	5 (4%)
T2, fat saturated, axial	2 (1.6%)
T2, other	8 (6.4%)
Unknown/unavailable	10
Slice thickness	
2 mm	1 (0.9%)
2.5 mm	1 (0.9%)
3 mm	109 (94%)
5 mm	5 (4.3%)
Unknown/Unavailable	19

Supplemental Table 2 Baseline cerebral MRI characteristics, by treatment group. *EPO=Erythropoietin. Gd+=Gadolinium contrast enhancing. MRI=Magnetic resonance imaging.*

	All patients with MRI at baseline				Subset with follow-up at month 6			
**Characteristic**	**N**	**EPO** N = 40[1]	**PLACEBO** N = 37[1]	**Statistic, P value**[2]	**N**	**EPO** N = 20[1]	**PLACEBO** N = 20[1]	**Statistic, P value**[2]
T2 infratentorial lesions, #	76			χ^2=6.66, P=.72	38			χ^2=0.00, P=1.0
0		31 (78%)	25 (69%)			16 (84%)	16 (84%)	
1–3		8 (20%)	10 (28%)			3 (16%)	3 (16%)	
4–6		1 (2.5%)	1 (2.8%)			0 (0%)	0 (0%)	
7+		0 (0%)	0 (0%)			0 (0%)	0 (0%)	
Median (IQR)		0.0 (0.0, 0.0)	0.0 (0.0, 1.0)	W = 677.0, P=.57		0.0 (0.0, 0.0)	0.0 (0.0, 0.0)	W = 182, P=.98
T2 juxtacortical lesions, #	76			χ^2=1.86, P=.60	38			χ^2=.49, P=.92
0		23 (59%)	22 (59%)			12 (63%)	10 (53%)	
1–3		11 (28%)	7 (19%)			4 (21%)	5 (26%)	
4–6		3 (7.7%)	6 (16%)			2 (11%)	3 (16%)	
7+		2 (5.1%)	2 (5.4%)			1 (5.3%)	1 (5.3%)	
Median (IQR)		0.0 (0.0, 1.0)	0.0 (0.0, 2.0)	W = 700, P=.80		0.0 (0.0, 1.0)	0.0 (0.0, 3.0)	W = 159, P=.48
T2 periventricular lesions, #	77			χ^2=3.21, P=.36	38			χ^2=4.62 P=.20
0		14 (35%)	15 (41%)			6 (32%)	8 (41%)	
1–3		17 (43%)	11 (30%)			9 (47%)	3 (16%)	
4–6		3 (7.5%)	7 (19%)			2 (11%)	4 (21%)	
7+		6 (15%)	4 (11%)			2 (11%)	4 (21%)	
Median (IQR)		2.0 (0.0, 3.0)	2.0 (0.0, 4.0)	W = 761, P=.83		2.0 (0.0, 3.0)	2.0 (0.0, 5.0)	W = 169, P=.74
T2 lesions, total #	75			χ^2=5.44, P=.14	38			χ^2= 2.32, P=.51
0		12 (31%)	13 (36%)			6 (32%)	7 (37%)	
1–3		15 (38%)	6 (17%)			7 (37%)	3 (16%)	
4–6		3 (7.7%)	7 (19%)			3 (16%)	4 (21%)	
7+		9 (23%)	10 (28%)			3 (16%)	5 (26%)	
Median (IQR)		3.0 (0.0, 6.0)	3.0 (0.0, 9.0)	W = 676, P=.78		3.0 (0.0, 5.0)	3.0 (0.0, 9.0)	W = 166, P=.67
Gd+ infratentorial lesions, #	74			χ^2=2.17, P=.34	37			χ^2=0.03, P=.87
0		38 (100%)	34 (94%)			18 (100%)	19 (100%)	
1–3		0 (0%)	1 (2.8%)			0 (0%)	0 (0%)	
4–6		0 (0%)	1 (2.8%)			0 (0%)	0 (0%)	
7+		0 (0%)	0 (0%)			0 (0%)	0 (0%)	

		All patients with MRI at baseline				Subset with follow-up at month 6		
**Characteristic**	**N**	**EPO** N = 40[1]	**PLACEBO** N = 37[1]	**Statistic, P value[2]**	**N**	**EPO** N = 20[1]	**PLACEBO** N = 20[1]	**Statistic, P value[2]**
Median (IQR)		0.0 (0.0, 0.0)	0.0 (0.0, 0.0)	W = 646, P=.15		0.0 (0.0, 0.0)	0.0 (0.0, 0.0)	W = 171, --[3]
Gd+ juxtacortical lesions, #	74			χ^2=2.75, P=.25	37			χ^2=0.00, P=1.00
0		34 (89%)	34 (94%)			17 (94%)	18 (95%)	
1–3		4 (11%)	1 (2.8%)			1 (5.6%)	1 (5.3%)	
4–6		0 (0%)	0 (0%)			0 (0%)	0 (0%)	
7+		0 (0%)	1 (2.8%)			0 (0%)	0 (0%)	
Median (IQR)		0.0 (0.0, 0.0)	0.0 (0.0, 0.0)	W = 716, P=.47	37	0.0 (0.0, 0.0)	0.0 (0.0, 0.0)	W = 172, P=1.0
Gd+ periventricular lesions, #	74			χ^2=.00, P=1.00	37			χ^2=0.27, P=.60
0		31 (82%)	30 (83%)			14 (78%)	17 (89%)	
1–3		7 (18%)	6 (17%)			4 (22%)	2 (11%)	
4–6		0 (0%)	0 (0%)			0 (0%)	0 (0%)	
7+		0 (0%)	0 (0%)			0 (0%)	0 (0%)	
Median (IQR)		0.0 (0.0, 0.0)	0.0 (0.0, 0.0)	W = 699, P=.83		0.0 (0.0, 0.0)	0.0 (0.0, 0.0)	W = 190, P=.38
Gd+ lesions, total #	74			χ^2=1.11, P=.78	37			χ^2=1.26, P=.53
0		30 (79%)	28 (78%)			14 (78%)	17 (89%)	
1–3		6 (16%)	5 (14%)			3 (17%)	1 (5.3%)	
4–6		2 (5.3%)	2 (5.6%)			1 (5.6%)	1 (5.3%)	
7+		0 (0%)	1 (2.8%)			0 (0%)	0 (0%)	
Median (IQR)		0.0 (0.0, 0.0)	0.0 (0.0, 0.0)	W = 672.5, P=.87		0.0 (0.0, 0.0)	0.0 (0.0, 0.0)	W = 190, P=.38
MRI Dissemination in Time	77	8 (20%)	9 (24%)	χ^2=.03, P=.86	38	4 (21%)	2 (11%)	χ^2=.20, P=.66
MRI Dissemination in Space	77	17 (43%)	19 (51%)	χ^2=.30, P=.58	38	8 (42%)	10 (53%)	χ^2=.11, P=.75

[1]n (%); Median (IQR)

[2]Pearson's Chi-squared test; Wilcoxon rank sum test

[3]P value could not be calculated

Supplementary Table 3: Baseline characteristics by time to treatment. "Early" refers to treatment that was initiated < 6 days from symptom onset. "Late" refers to treatment that was initiated ≥ 6 days from symptom onset. *Gd+=Gadolinium enhancing. EPO=Erythropoietin. MRI=Magnetic resonance imaging. IQR=Interquartile range. MP=Methylprednisolone.*

	All patients with MRI at baseline				Subset with MRI follow-up at month 6			
**Characteristic**	**N**	**Early treatment,** N = 37[1]	**Late treatment,** N = 40[1]	**Statistic, P value**[2]	**N**	**Early treatment,** N = 20[1]	**Late treatment,** N = 20[1]	**Statistic, P value**[2]
Demographics								
Sex	77			χ^2=.00, P=.99	40			χ^2=.00, P=1.00
Female		26 (70%)	27 (67%)			13 (65%)	14 (70%)	
Male		11 (30%)	13 (33%)			7 (35%)	6 (30%)	
Age, years	77	28.0 (23.0, 37.0)	30.0 (27.0, 36.3)	W= 599, P=.15	40	28.0 (25.0, 35.5)	30.0 (27.0, 37.8)	W=160, P=.28
Treatment								
Assigned treatment	77			χ^2=.34, 0.4	40			χ^2=.90, P=.34
EPO + MP		21 (57%)	19 (48%)			8 (40%)	12 (60%)	
Placebo + MP		16 (43%)	21 (53%)			12 (60%)	8 (40%)	
Time from symptom onset to treatment, days	77	4.0 (3.0, 4.0)	7.0 (6.0, 8.0)	W=.00, **P<0.001**	40	3.5 (3.0, 4.0)	7.0 (6.0, 9.0)	W=.00, **P<0.001**
Cerebral MRI								
T2 infratentorial lesions, #	76			χ^2=.02, P=.99	38			χ^2=.00, P= 1.00
0		27 (73%)	29 (74%)			15 (83%)	17 (85%)	
1-3		9 (24%)	9 (23%)			3 (17%)	3 (15%)	
4-6		1 (2.7%)	1 (2.6%)			0 (0%)	0 (0%)	
7+		0 (0%)	0 (0%)			0 (0%)	0 (0%)	
Median (IQR)		0 (0–1)	0 (0–1)	W=730, P=.91		0 (0–0)	0 (0–0)	W=179.5, P=1.00
T2 juxtacortical lesions, #	76			χ^2=3.15, P=.37	38			χ^2=.02, P=.61
0		19 (51%)	26 (67%)			9 (50%)	13 (65%)	
1-3		12 (32%)	6 (15%)			6 (33%)	3 (15%)	
4-6		4 (11%)	5 (13%)			2 (11%)	3 (15%)	
7+		2 (5.4%)	2 (5.1%)			1 (5.6%)	1 (5.0%)	
Median (IQR)		0 (0–2)	0 (0–1)	W=818, P=.26		0.5 (0–2)	0 (0–2)	W=200, P=.52
T2 periventricular lesions, #	77			χ^2=3.01, P=.39	38			χ^2=3.86, P=.28
0		15 (41%)	14 (35%)			6 (33%)	8 (40%)	
1-3		10 (27%)	18 (45%)			5 (28%)	7 (35%)	
4-6		6 (16%)	4 (10%)			5 (28%)	1 (5.0%)	
7+		6 (16%)	4 (10%)			2 (11%)	4 (20%)	
Median (IQR)		1 (0–4)	2 (0–3)	W=731.5, P=.93		1.5 (0–3.5)	2 (0–3.5)	W=191, P=.75
T2 lesions, total #	75			χ^2=0.95, P=.81	38			χ^2=.73, P=.87
0		12 (32%)	13 (34%)			5 (28%)	8 (40%)	
1-3		10 (27%)	11 (29%)			5 (28%)	5 (25%)	
4-6		4 (11%)	6 (16%)			4 (22%)	3 (15%)	
7+		11 (30%)	8 (21%)			4 (22%)	4 (20%)	

	All patients with MRI at baseline				Subset with MRI follow-up at month 6			
**Characteristic**	**N**	**Early treatment,** N = 37[1]	**Late treatment,** N = 40[1]	**Statistic, P value**[2]	**N**	**Early treatment,** N = 20[1]	**Late treatment,** N = 20[1]	**Statistic, P value**[2]
Median (IQR)		1 (0–4)	2 (0–3)	W=731, P=.93		2.5 (0–6)	3 (0–6)	W=192, P=.73
Gd+ infratentorial lesions, #	74			χ^2=1.84, P=.40	37			χ^2=.03, P=.87
0		35 (100%)	37 (95%)			18 (100%)	19 (100%)	
1-3		0 (0%)	1 (2.6%)			0 (0%)	0 (0%)	
4-6		0 (0%)	1 (2.6%)			0 (0%)	0 (0%)	
7+		0 (0%)	0 (0%)			0 (0%)	0 (0%)	
Median (IQR)		0 (0–1)	0 (0–0)	W=648, P=.18		0 (0–0)	0 (0–0)	W=171.0, --[3]
Gd+ juxtacortical lesions, #	74			χ^2=3.12, P=.21	37			χ^2=.59, P=.44
0		31 (89%)	37 (95%)			16 (89%)	19 (100%)	
1-3		4 (11%)	1 (2.6%)			2 (11%)	0 (0%)	
4-6		0 (0%)	0 (0%)			0 (0%)	0 (0%)	
7+		0 (0%)	1 (2.6%)			0 (0%)	0 (0%)	
Median (IQR)		0 (0–0)	0 (0–0)	W=724, P=.35		0 (0–0)	0 (0–0)	W=190, P=.15
Gd+ periventricular lesions, #	74			χ^2=.68, P=.41	37			χ^2=1.99, P=.16
0		27 (77%)	34 (87%)			13 (72%)	18 (95%)	
1-3		8 (23%)	5 (13%)			5 (28%)	1 (5.3%)	
4-6		0 (0%)	0 (0%)			0 (0%)	0 (0%)	
7+		0 (0%)	0 (0%)			0 (0%)	0 (0%)	
Median (IQR)		0 (0–0)	0 (0–0)	W=759, P=.22		0 (0–1)	0 (0–0)	W=210.5, P=0.64
Gd+ lesions, total #	74			χ^2=2.16, P=.54	37			χ^2=3.78, P=.15
0		27 (77%)	31 (79%)			13 (72%)	18 (95%)	
1-3		5 (14%)	6 (15%)			3 (17%)	1 (5.3%)	
4-6		3 (8.6%)	1 (1.6%)			2 (11%)	0 (0%)	
7+		0 (0%)	1 (2.6%)			0 (0%)	0 (0%)	
Median (IQR)		0 (0–0)	0 (0–0)	W=703, P=.77		0 (0–1)	0 (0–0)	W=210.5, P=.06
Classification								
MRI dissemination in space	77	18 (49%)	18 (45%)	χ^2=.01, P=.93	38	10 (56%)	8 (40%)	χ^2=.40, P=.53
MRI dissemination in time	77	9 (24%)	8 (20%)	χ^2=.03, P=.86	38	5 (28%)	1 (5.0%)	χ^2=2.18, P=.14
Optic nerve MRI								
Cross-sectional area, mm^2	55	5.0 (4.0, 7.0)	6.0 (4.0, 7.8)	W=348.5, P=.66	30	5.0 (4.0, 7.0)	6.0 (4.0, 7.0)	W=97.0, P=.58
Cross-sectional area, inter-eye ratio	54	1.0 (1.0, 1.2)	1.0 (1.0, 1.1)	W=377.5, P=.75	30	1.0 (1.0, 1.0)	1.0 (1.0, 1.0)	W=111.0, P=1.0
T2 lesions								
Lesion present	70	25 (69%)	27 (79%)	χ^2 = 0.46, P=0.50	36	13 (72%)	17 (94%)	χ^2 = 1.80, P=0.18
Lesion length, mm	52	9.0 (9.0, 12.0)	9.0 (6.0, 12.0)	W=368.5, P=.56	30	9.0 (9.0, 12.0)	9.0 (6.0, 12.0)	W=116.5, P=.81
Gd+ lesions								
Lesion present	62	17 (57%)	23 (72%)	χ^2 = 0.97, P=0.33	35	9 (53%)	13 (72%)	χ^2 = 0.69, P=0.41
Lesion length, mm	40	9.0 (6.0, 12.0)	9.0 (7.5, 10.5)	W=169.0, P=.46	22	9.0 (6.0, 9.0)	9.0 (6.0, 9.0)	W=54.0, P=.78

[1]n (%); Median (IQR)
[2]Pearson's Chi-squared test; Wilcoxon rank sum test
[3]P value could not be calculated

Supplementary Table 4. Outcomes of cerebral white matter MRI lesions at month 6, by time to treatment. "Early" refers to treatment that was initiated < 6 days from symptom onset. "Late" refers to treatment that was initiated ≥ 6 days from symptom onset. *Gd+=Gadolinium enhancing. MRI=Magnetic resonance imaging.*

Characteristic	N	Early treatment, N = 20[1]	Late treatment, N = 20[1]	Statistic, P value[2]
Change in number of T2 infratentorial lesions	38			χ2=2.01, P=.37
-1 to -3		0 (0%)	1 (5.0%)	
0		17 (94%)	19 (95%)	
1		1 (5.6%)	0 (0%)	
Median (IQR)		0 (0, 0)	0 (0, 0)	W=189, P=.50
Change in number of T2 juxtacortical lesions	37			χ2=3.51, 0.32
-1 to -3		2 (11%)	2 (11%)	
0		13 (72%)	17 (89%)	
1–3		2 (11%)	0 (0%)	
4–6		1 (5.6%)	0 (0%)	
Median (IQR)		0 (0, 0)	0 (0, 0)	W=197, P=.27
Change in number of T2 periventricular lesions	38			χ2=3.44, P=.49
≤ -7		0 (0%)	1 (5.0%)	
-1 to -3		3 (17%)	1 (5.0%)	
0		13 (72%)	17 (85%)	
1–3		1 (5.6%)	1 (5.0%)	
4–6		1 (5.6%)	0 (0%)	
Median (IQR)		0 (0, 0)	0 (0, 0)	W=181, P=1.0
Change in total number of T2 lesions	37			χ2=2.84, P=.59
≤ -7		0	1 (5.3%)	
-1 to -3		3 (17%)	2 (11%)	
0		12 (67%)	15 (79%)	
1–3		2 (11%)	1 (5.3%)	
7+		1 (5.6%)	0 (0%)	
Median (IQR)		0 (0, 0)	0 (0, 0)	W= 189, P=.50
Change in number of Gd+ infratentorial lesions	36			
0		18 (100%)	18 (100%)	χ2=0.00, P=1.0
Median (IQR)		0 (0–0)	0 (0–0)	W=199, --[3]
Change in number of Gd+ juxtacortical lesions	36			χ2= 1.33, P=.51
-1 to -3		1 (5.6%)	2 (11%)	
0		16 (89%)	16 (89%)	
1–3		1 (5.6%)	0 (0%)	
Median (IQR)		0 (0, 0)	0 (0, 0)	W=179, P=.34
Change in number of Gd+ periventricular lesions	36			χ2= 6.53, **P=.04**
-1 to -3		0 (0%)	1 (5.6%)	
0		13 (72%)	17 (94%)	
1–3		5 (28%)	0 (0%)	
Median (IQR)		0 (0, 1)	0 (0, 0)	W=214, **P=0.01**
Change in total number of Gd+ lesions	36			χ2= 4.53, P=.21
-1 to -3		1 (5.6%)	1 (5.6%)	
0		13 (72%)	17 (94%)	
1–3		3 (17%)	0 (0%)	
4–6		1 (5.6%)	0 (0%)	
Median (IQR)		0 (0, 0)	0 (0, 0)	W=197, P=.01

Characteristic	N	Early treatment, N = 20[1]	Late treatment, N = 20[1]	Statistic, P value[2]
MRI dissemination in time	40	7 (35%)	3 (15%)	P=.14
MRI dissemination in space	40	11 (55%)	9 (45%)	P=.50

[1]n (%)

[2]Pearson's Chi-squared test; Fisher's exact test

[3]P value could not be calculated

Supplementary Table 5. Outcomes of optic nerve MRI at month 6, by time to treatment. “Early” refers to treatment that was initiated < 6 days from symptom onset. “Late” refers to treatment that was initiated ≥ 6 days from symptom onset. *Gd+=Gadolinium enhancing. MRI=Magnetic resonance imaging.*

**Characteristic**	**N**	**Early treatment**, N = 20[1]	**Late treatment**, N = 20[1]	**Statistic, P value**[2]
All patients				
Cross-sectional area, mm^2	25	4.00 (4.00, 5.00)	5.50 (4.00, 7.25)	W=59.5, P=0.3
Change in cross-sectional area, mm^2	21	-1.00 (-1.00, 0.00)	-0.50 (-1.25, 0.00)	W=51.0, P=0.9
Cross-sectional area, inter-eye ratio	24	.80 (.71, 1.00)	1.00 (.80, 1.00)	W=55.5, P=.35
Patients with T2 lesions at baseline				
T2 lesion present	25	9 (82%)	13 (93%)	χ^2=.05, P=.47
T2 lesion length, mm	25	9.0 (9.0, 15.0)	9.0 (6.0, 9.0)	W=82.0, P=.10
Change in T2 lesion length, mm	25	0.0 (0.0, 3.0)	0.0 (-3.0, 3.0)	W=91.5, P=.43
Patients with Gd+ lesions at baseline				
Gd+ lesion present	15	3 (43%)	3 (38%)	χ^2=0.0, P=1.0
Gd+ lesion length, mm	6	9.0 (3.0, 9.0)	6.0 (6.0, 18.0)	W=4.0, P=1.0
Change in Gd+ lesion length, mm	14	-6.0 (-10.0, 0.0)	-7.5 (-12.0, -6.0)	W=28.0, P=.64

[1]Median (IQR)
[2]Wilcoxon rank sum test, Chi^2 test

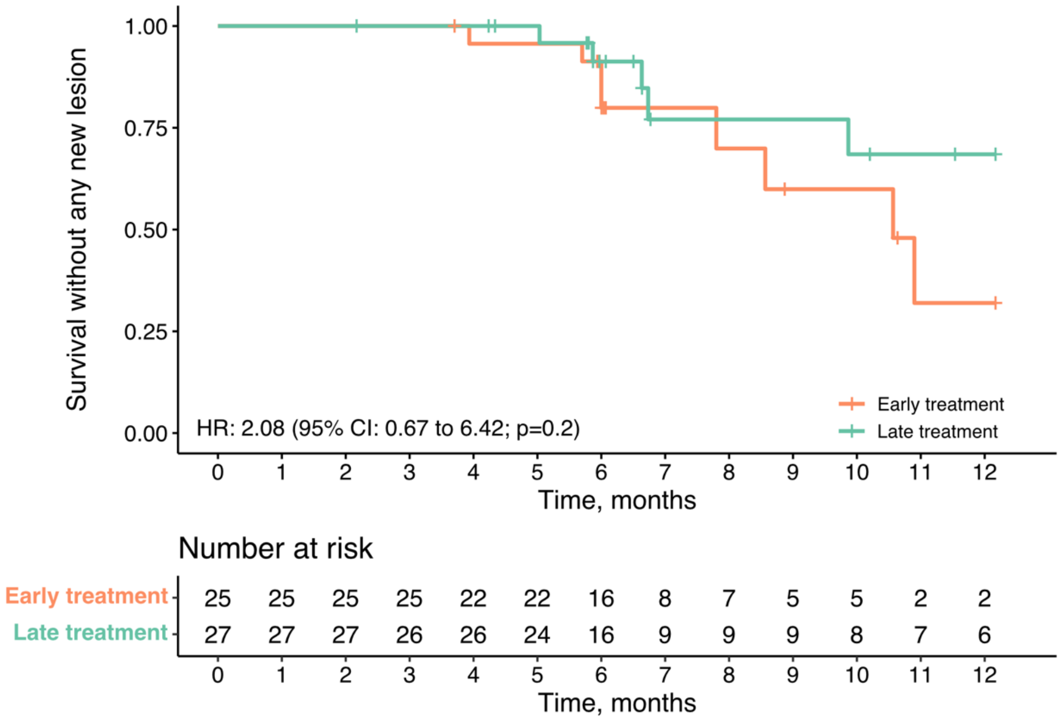

Supplementary Figure 1. Survival without any new cerebral white matter lesion, by time to treatment. Early" refers to treatment that was initiated < 6 days from symptom onset. "Late" refers to treatment that was initiated ≥ 6 days from symptom onset. Staatistical Analysis was by a Cox proportional hazards model. *HR=Hazard ratio. CI=Confidence interval.*
